# Differential Effects of Neurotensin NTS1 and NTS2 Receptors on Locomotion

**DOI:** 10.1002/brb3.71060

**Published:** 2025-11-14

**Authors:** Misty D. Smith, Elizabeth Jill Dahle, Annette E. Fleckenstein, Glen R. Hanson

**Affiliations:** ^1^ Pharmacology and Toxicology College of Pharmacy Salt Lake City Utah USA; ^2^ School of Dentistry University of Utah Salt Lake City Utah USA

## Abstract

**Introduction:**

Neurotensin (NT) is an endogenous neuropeptide with diverse central and peripheral effects, particularly as related to modulation of central nervous system dopaminergic activity. For example, interactions between dopamine and NT have been associated with the motivation to use, and the motor consequences of drugs abuse, including nicotine. However, the relative contribution of the two subtypes of cell surface G‐protein coupled NT receptors (NTS1 and NTS2) to dopamine‐related drug‐induced effects is unclear.

**Methods:**

We investigated the locomotor behavior and exploratory drive of C57BL/6J mice deficient in either NTS1 (NTS1 −/−) or NTS2 (NTS2 −/−) compared to wild‐type C57BL/6J (WT +/+) mice in an open‐field. In addition, the effect of nicotine on locomotion and intra‐session habituation to a novel open field was compared in each of these genetic strains.

**Results:**

When compared to WT (+/+) mice, the results demonstrated less intra‐session habituation across time (i.e., less accommodation (as assessed by distanced travelled, horizontal activity, and vertical activity) in mice deficient in the NTS1 receptor. In contrast, mice deficient in the NTS2 receptor accommodated more rapidly. Nicotine injection reduced all three parameters of locomotor activity in WT (+/+) and NTS1 (−/−) mice. In contrast to effects in both WT (+/+) and NTS1 (−/−) mice, NIC exposure had a negligible effect on TD in the NTS2 (−/−) mice.

**Conclusion:**

These results suggest opposing effects of the NTS1 and NTS2 receptor subtypes in modulating natural and nicotine‐induced dopaminergic transmission and consequent locomotor behavior.

## Graphical abstract

The present data demonstrate that the absence of NTS1 and NTS2 receptors differentially impacts habituation in a novel open field. We speculate that NIC treatment, by increasing NT release, activates NTS1 receptors and causes these mice to habituate more rapidly than WT mice in a novel open field.

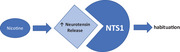

## 1 Introduction

Substances associated with the development of substance use disorders (SUDs) linked to drug dependence behavior include psychostimulants such as amphetamine, cocaine, and nicotine (NIC). While the pharmacological mechanisms related to SUDs associated with these compounds are distinct (e.g., monoamine releasers and uptake blockers, and receptor ligand agonists, respectively), these have some common features that are linked to their central nervous system effects such as SUD emergence and their potential treatment strategies. Such mechanistic conclusions have been supported by reports that the neuropeptide, neurotensin (NT), is linked with the modulation of the extrapyramidal/mesolimbic dopaminergic (DAergic) systems connected with expression of hyperactivity and locomotor sensitization caused by the abuse of these substances (Fredrickson et al. 2014, 2003). The present research builds on these findings by employing selective gene‐knockout technology in mice to test the impact of selective removal of the two principal NT receptors (NTS1 and NTS2) on NIC‐induced locomotor/sensitization behaviors.

Exploratory locomotor behavior of mice in a novel open‐field paradigm has been used to assess motivated behavior and habituation. While systemically administered NT per se has no effect on locomotion in most studies (see Elliott et al. 1986; Elliott and Nemeroff 1986, and references therein), intraperitoneal (i.p.) administration of the NTS1‐selective agonist PD149163 attenuates locomotor activity in both a novel environment and in the home cage (Vadnie et al. 2014). Additionally, central NT injection has complex, site selective effects on locomotion (Kalivas et al. 1982, 1984).

Although NT modulates DAergic neurotransmission, the relative contribution of the two principal central receptor subtypes, NTS1 and NTS2, to exploratory drive in a novel open field and in the response to psychostimulants such as NIC is unclear. The present study addressed this issue by comparing C57BL/6J mice deficient in either NTS1 (NTS1 (−/−)) or NTS2 (NTS2 (−/−)) to wild‐type (WT), littermate C57BL/6J (WT (+/+)) mice in a novel open field following exposure.

## 2 Methods

Male and female mice (8–12 weeks of age; *n* = 8/group) were generated from mice obtained from the Wada laboratory (Maeno et al. 2004). Owing to littermate availability, three male and five female WT, five male and three female NTS1 (−/−) and two female and six male NTS2 (−/−) were utilized. At the time of experiments, NTS1‐deficient mice had been back‐crossed to WT C57BL/6J at least 39 times and NTS2‐deficient mice had been back‐crossed to WT C57BL/6J at least 23 times. For each receptor subtype, male and female heterozygous progeny for each subtype was then crossed to generate homozygous NTS1 and homozygous NTS2 deficient mice, respectively. Genotypes of mice were confirmed with PCR of total genomic DNA isolated from tail samples using the following primers: NTS1 WT PCR, NTS1 exon 2 forward: 5‐GTT AAC ACC TTC ATG TCC TTC CTG‐3 and NTS1 exon 2 reverse: 5‐TAC GTA AGA CGA GGA CTC CAT GGC G‐3 (195 base pair product); NTS1 mutant PCR, NTS1 neo forward: 5‐GGA TCG GCC ATT GAA CAA GAT GG‐3 and NTS1 neo reverse: 5‐CTT CAG CAA TAT CAC GGG TAG CC‐3 (696 base pair product); NTS2 WT and mutant PCR, NTS2 exon 1 forward: 5‐ATC AGG CCA CCT CGA GAC AGA GAT G‐3, NTS2 exon 1 reverse: 5‐GCA CGA AGT AAT AGC CAC GAC AGC C‐3 and NTS2 neo forward: 5‐GGC TAC CCG TGA TAT TGC TGA AGA G‐3 (WT, 350 base pair product and mutant, 620 base pair product). Genotypes of mice were confirmed with PCR using the primers described by Maeno et al. (2004). All experiments were approved by the University of Utah's Institutional Animal Care and Use Committee and handled consistent with recommendations from the *Guide for the Care and Use of Laboratory Animals*.

**FIGURE 1:**
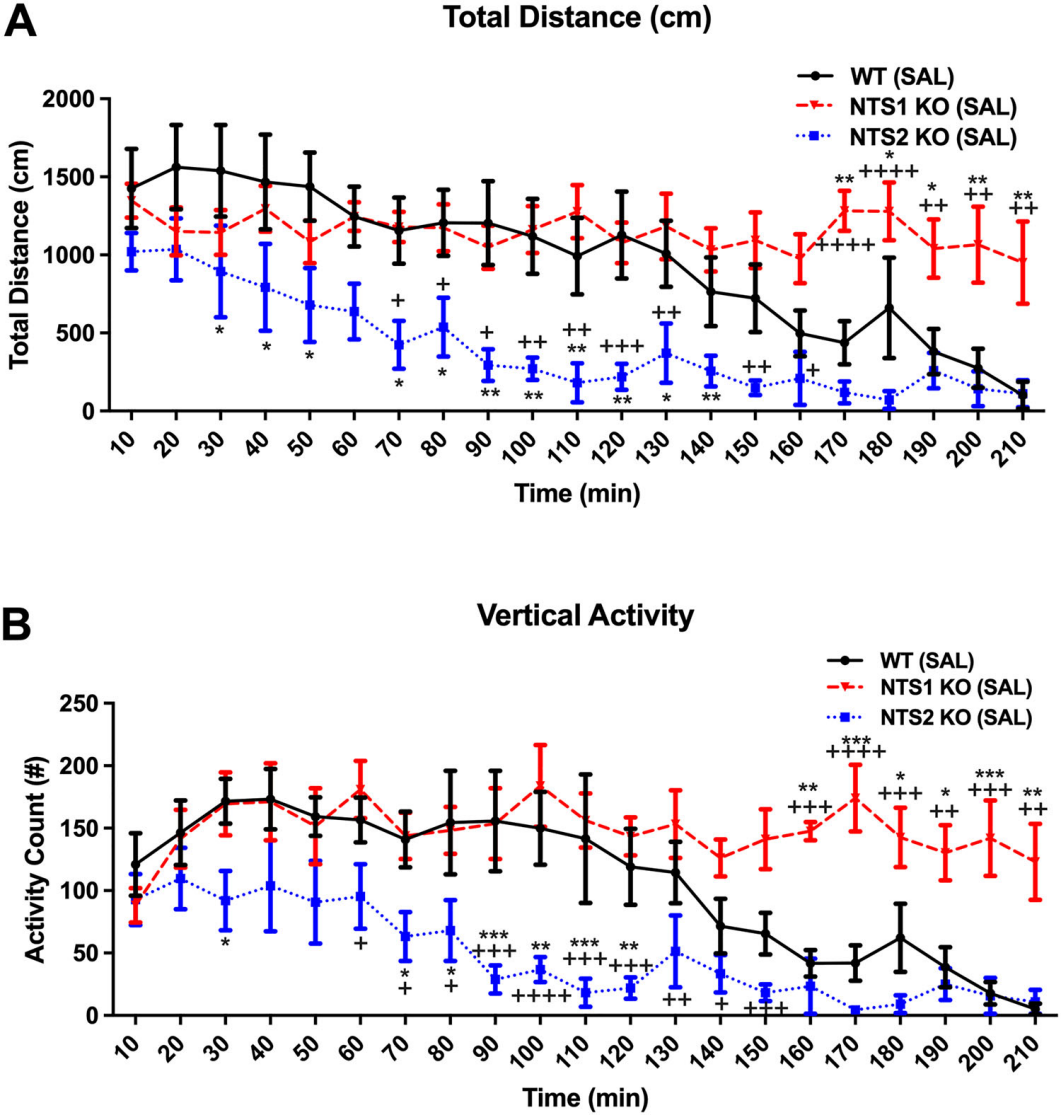
Differential effects of genotype and time on (A) total distance (TD) travelled and (B) vertical activity counts (VAC) in the open field were compared in NTS1‐ and NTS2‐KO versus age matched WT mice. Data were compared statistically using two‐way ANOVA with Tukey's post hoc multiple comparisons test (GraphPad Prism 10.4.1 for Mac, GraphPad, La Jolla, CA, USA). Differences were considered statistically significant if *p* ≤ 0.05. In this figure, significant differences compared to WT saline treated mice are indicated as *p < 0.05, **p < 0.01, ***p < 0.001, whereas statistical differences between NTS1 and NTS2 KO groups are shown as ^+^ *p* < 0.05, ^++^ *p* < 0.01, ^+++^ *p* < 0.001 or ^++++^ *p* < 0.0001.

**FIGURE 2:**
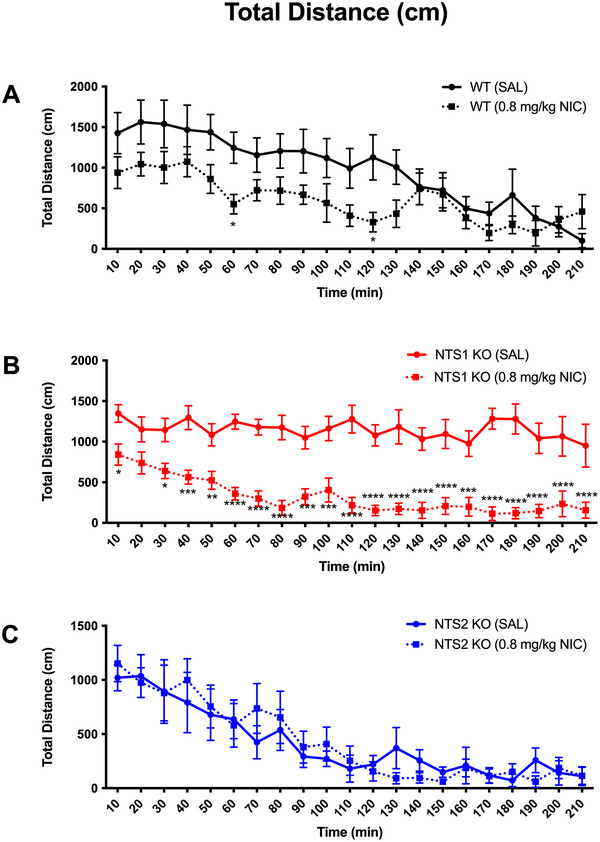
Differential effect of 0.8 mg/kg, i.p., NIC on total distance travelled in WT (A), NTS1 KO (B), and NTS2 KO mice (C). Data were compared statistically using two‐way ANOVA with Tukey's post hoc multiple comparisons test (GraphPad Prism 10.4.1 for Mac, GraphPad, La Jolla, CA, USA). Differences were considered statistically significant if *p* ≤ 0.05. Significant differences compared to saline treated mice are indicated as *p < 0.05, **p < 0.01, ***p < 0.001. Mice (*n* = 8/group) were utilized as detailed in Section 2.

Groups of mice from each genotype received nicotine at 0.4 mg/kg, i.p. (not shown) or 0.8 mg/kg, i.p. (NIC, Sigma, St. Louis, MO, USA) or an equivalent volume of 0.9% saline vehicle (i.p.). Exploratory locomotor behavior was evaluated using an AccuScan activity monitoring system (OmniTech Instruments Inc., Columbus, OH, USA) and the Fusion software. Mice were allowed to acclimate in their home cages in the testing room for a minimum of 30 min prior to drug or vehicle administration. After injection, each mouse was placed into one of eight clear acrylic open field chambers (each 40 cm × 40 cm × 30 cm). Locomotor parameters were recorded across 21 consecutive 10‐min epochs to assess each strain's habituation to a novel open‐field environment. Experiments were performed between 0800 and 1400 h. Total distance travelled (TD), horizontal activity (HAC), and vertical activity (VAC) were compared statistically using two‐way analysis of variance with Tukey's post hoc multiple comparisons test (GraphPad Prism 10.4.1 for Mac; GraphPad, La Jolla, CA, USA). Differences were considered statistically significant if *p* ≤ 0.05. Statistical differences compared to WT saline treated mice were indicated as *p < 0.05, **p < 0.01, ***p < 0.001, whereas statistical differences between NTS1 and NTS2 KO groups in Figure 1 are indicated as ^+^ *p* < 0.05, ^++^ *p* < 0.01, ^+++^ *p* < 0.001, or ^++++^ *p* < 0.0001. For Figure 2, significant differences compared to saline treated mice are indicated as *p < 0.05, **p < 0.01, ***p < 0.001.

## 3 Results

Results presented in Figure 1A revealed that TD traveled by NTS2 (−/−) mice was reduced, particularly during the first 120 min of testing (i.e., a time period referred to herein as “phase I”) compared to saline‐treated WT mice. In contrast, NTS1 KO mice displayed increased TD travelled compared to WT mice, particularly beginning at approximately 120 min post placement in the chamber (i.e., a period referred to herein as Phase II). Similar changes in VAC (Figure 1B) and (HAC; data not shown) were observed in saline‐treated NTS1 (−/−) and NTS2 (−/−) relative to WT mice. Based on these data, intra‐session habituation appears to be sensitive to NT systems in a counter‐regulatory fashion: thus, NTS1‐deficient mice had less intra‐session habituation across time (especially in phase II), whereas NTS2‐deficient mice habituated faster, than similarly treated WT mice. It appears that the predominance of these receptors’ effects varies in a time‐dependent fashion. That is, in “phase I,” NTS2 are activated in WT mice, and thus habituation is minimal, while later in “phase II,” NTS1 activation dominates causing rapid habituation.

Of note, increased locomotion has been reported in both NTS1 (−/−) and NTS2 (−/−) mice (Liang et al. 2010). This difference is most likely due to variance in experimental design, as our laboratory acclimated mice to the room in their home cage and then evaluated mice from the initial introduction to the novel open field, whereas Liang et al. acclimated the mice to the activity chamber for 2 h prior to recording.

Our laboratory reported that NIC reduces NT‐like immunoreactivity in the VTA, a midbrain region implicated in the locomotor and rewarding effects of NIC and other addictive drugs (Alburges et al. 2009, 2007). Because our KO data herein indicate that NT receptors are involved in habituation to the novel open field, we examined the impact of NIC in the patterns of locomotion in NTS1 (−/−) and NTS2 (−/−) mice.

The results presented in Figure 2A demonstrate that NIC exposure decreased TD travelled in WT mice in phase I compared with saline‐treated controls (with WT‐control data repeated from Figure 1A), with significance achieved at the 60‐ and 120‐min epochs. Similar effects were observed for HAC and VAC (data not shown).

Figure 2B demonstrates that NIC resulted in more substantial and sustained decrease in TD throughout the entire 210‐min observation in NTS1 (−/−) mice relative to saline controls (with NTS1 (−/−) data repeated from Figure 1A). Similar effects on HAC and VAC were observed in NTS1 (−/−) mice (data not shown). We speculate that NIC treatment, by increasing NT release, activates NTS1 receptors and causes these treated mice to habituate more rapidly than WT mice. These data are consistent with previous findings that intracerebroventricular NT administration induces hypolocomotion in WT, but not in NTS1 (−/−) mice (Pettibone et al. 2002; Remaury et al. 2002).

In contrast to effects in NTS1 (−/−) mice, NIC exposure had little effect on TD in the NTS2 (−/−) mice (Figure 2C; with NTS2 (−/−) data repeated from Figure 1A). Similarly, NIC had little or no effect on HAC and VAC in NTS2 (−/−) mice (data not shown). Thus, we speculate that NIC‐mediated decreases in locomotor activity are NTS2‐independent.

## 4 Discussion

NT modulates dopaminergic neurotransmission throughout the brain and body, including the nigrostriatal and the mesocorticolimbic pathways (Kitabgi 1989; Nemeroff 1986). It induces hypolocomotion in WT, but not NTS1 (−/−) mice, following intracerebroventricular administration (Pettibone et al. 2002; Remaury et al. 2002). However, little is known about the influence of the two G‐coupled receptor subtypes of the NT receptor on basal locomotor behavior in a novel open field or in the locomotor response to an acute exposure of NIC, a stimulant that increases locomotor activity via DAergic mechanisms. With regard to the former, no significant difference was observed between saline‐treated WT, NTS1 (−/−), or NTS2 (−/−) mice in baseline locomotor activity in the novel open‐field chamber during the first 10‐min epoch after cage placement. This result is consistent with the findings by Remaury et al. (2002), of similar basal locomotor activity in NTS1 (−/−) mice immediately after placement in the open field; however, these observations lasted only 30 min and did not evaluate intra‐session habituation across longer amounts of time as mice continued to explore and then accommodate to the novel open field. Our data, particularly in the NTS1 (−/−) mice, demonstrate that the exploratory phase exceeds the initial 30 min of observation and suggest that loss of the NTS1 receptor impairs accommodation as a novel site becomes familiar. In contrast, Liang et al. (2010) observed increased locomotor activity in NTS1 (−/−) and NTS2 (−/−) mice. However, the increased baseline activity reported by Liang is most likely the result of differences in experimental design, since our laboratory evaluated mice from the initial introduction to the novel open field, whereas Liang et al. acclimated the mice to the activity chamber for 2 h prior to recording, adding an additional hour of locomotor activity in the familiar chamber. In female mice, knocking out the NTS2 leads to less baseline locomotor activity comparted to WT mice. Interestingly, this effect is not observed in male mice (Feifel et al. 2010).

Previous studies have dem^1996^onstrated that NIC or smoking increases DA release from the nucleus accumbens (Benowitz, 2010; Berrendero et al. 2005; DiChiara 2000; Tizabi et al. 2007; Wonnacott et al. 2005). With regard to the effects of an acute exposure to NIC, our laboratory previously reported that NIC increases NT release, which in turn inhibits DA release through a D2 receptor‐mediated mechanism (Alburges et al. 2007). Because activation of the NTS1 receptor inhibits DA transmission via interactions with dopamine D2 receptors (Binder et al. 2001), and elevated D2 receptor activity increases locomotor activity (Kelly et al. 1998; Klinker et al. 2013), it has been suggested that NT reduces locomotion through inhibition of D2 receptor function (Vadnie et al. 2014). In the present experiments, we observed differences in the within‐session habituation to a novel open field between WT, NTS1 (−/−) and NTS2 (−/−) mice that suggest the NTS1 and NTS2 receptor subtypes have counter‐regulatory roles on the NT‐DAergic interactions. We found normal habituation to the novel open field in WT mice throughout the 210‐min observation period. We hypothesize that normally when introduced to the novel space, the stimulatory drive to explore eventually increases release of NT and stimulation of NTS2 receptors. Our findings suggest that in WT mice, NTS2 receptor stimulation inhibits NTS1 receptor function, thus increasing DA and driving increased locomotor exploration of the novel space. As the novelty begins to wear off, activation of NTS2 receptors diminishes, and NTS1‐mediated inhibition of DA reduces locomotor exploration of what is becoming familiar space. In the NTS2 (−/−) mice, the NTS1 receptors work unopposed, resulting in even faster within‐session accommodation as shown in Figure 1. Conversely, we observed delayed within‐session habituation (delayed accommodation) to the novel open field in the NTS1‐deficient mice, an effect that was reversed by the i.p. administration of nicotine (0.8 mg/kg, i.p.). We hypothesize that when NTS1‐deficient mice are first exposed to the novel open field, the stimulatory drive to explore the field may increase NT activation of the NTS2 receptors unopposed by NTS1 receptors, thus increasing DA levels and interfering with the normal accommodation response. If this hypothesis is correct, the administration of NIC in the NTS1‐deficient mice likely works through a non‐NTS‐mediated inhibitory mechanism, such as GABA, to control DA release and restore habituation.

Throughout these studies, it is important to appreciate that the elimination of NT receptors may have had development influence on other relevant systems that can impact behavioral response. This is a potential limitation of using knockout mice, although there is a significant literature supporting the use of NT receptor‐deficient mice for pharmacological and physiological studies. Of note, constitutive knockout lines, including NTS1(−/−) and NTS2 (−/−), exhibit developmental and compensatory changes that impact cell and circuit function and are not restricted to the deleted gene. For example, NTS1 (−/−) mice display elevated DAergic signaling and increased locomotor activity, while NTS1 antagonists do not cause the same effects indicating a disassociation between the developmental knockout phenotype and regular signaling via NTS1 (Liang et al. 2010; Perez‐Bonilla et al. 2022). Based on the findings described above, the present study suggests that the NTS1 subtype of the NT system facilitates, whereas NTS2 subtype inhibits, intra‐session habituation of locomotor activity in a novel environment as the mice accommodate to a novel open field. We speculate that NIC treatment, by increasing NT release, activates NTS1 receptors and causes these treated mice to habituate more rapidly than WT mice. Mechanistic studies underlying these effects, including evaluating the impact of selective NT antagonist, are warranted.

## Author Contributions

**Misty D. Smith**: conceptualization, data curation, formal analysis, investigation, methodology, project administration, resources, software, supervision, writing–original draft, writing–review and editing. **Elizabeth Jill Dahle**: . **Annette E. Fleckenstein**: data curation, formal analysis, investigation, project administration, resources, supervision, writing–original draft, writing–review and editing. **Glen R. Hanson**: conceptualization, data curation, formal analysis, funding acquisition, methodology, project administration, resources, software, supervision, visualization, writing–original draft, writing–review and editing.

## Funding

Funding was received from Glen Hanson NIDA DA000378, NIDA DA031883.

## Peer Review

The peer review history for this article is available at https://doi.org/10.1002/brb3.71060.

## Data Availability

The data that support the findings of this study are available from the corresponding author upon reasonable request.
